# The Biophysics of Visual Edge Detection: A Review of Basic Principles

**DOI:** 10.7759/cureus.11218

**Published:** 2020-10-28

**Authors:** Hassan Kesserwani

**Affiliations:** 1 Neurology, Flowers Medical Group, Dothan, USA

**Keywords:** vision disturbance, vision screening, mathematics, medical biophysics

## Abstract

The mathematization of nature is an age-old concept. The Greeks sought harmony in the celestial spheres. The Arab geometers constructed a spherical geometry of the heavens. Later, Galileo Galilei arithmetized kinematics. As the centuries advanced, polymaths like the Dutchman Christiaan Huygens applied more advanced mathematics in order to understand natural phenomena. It was not until the turn of the twentieth century that a more comprehensive mathematical approach to understanding biological phenomena was sought by D’Arcy Wentworth Thompson.

This leads us to our current review of the biophysics of visual edge detection. This is an unfolding saga of stunning experimental revelations in unison with an underlying mathematical edifice. The concept of visual contrast is a fundamental idea in order to understand the phenomenon of visual edge detection. We begin with contrast visual testing and the development of frequency tuning curves, which provided an insight into the multi-channel processing of selective spatial frequencies by the visual cortex. The single-cell recordings from the simple cells of the cat visual cortex unfolded the gamma distribution curves of different neuronal firing frequencies for different spatial frequencies. The theoretical construction of the convolution of Gabor wavelets with stimulus intensity goes hand-in-hand with the experimental observation of the separation of simple visual cortical cells into even and odd functions, a spectacular finding.

In this review, we march the reader through the mathematical basics and the pathophysiologic correlates. Beginning with a simple Fourier analysis of a square wave, Weber’s biophysical law, and the gamma distribution of contrast tuning curves, we gradually introduce Fourier transforms, the uncertainty principle of waveform analysis, the basics of wavelet theory, Gabor elementary signals and transforms, the concepts of coherence, and Weyl group representation theory. Group theory provides the symmetry operations necessary to preserve the fidelity of an image as it travels from the retina and cascades up the visual cortex. Unitary operators that allow a retinal displacement of an image to be reflected by a similar displacement in the visual cortex is also a fundamental principle. Along the way, we encounter the Convolution Theorem of Fourier transforms, which is critical in constructing a visual percept. We intermittently interject relevant clinical data as we unpack the mathematical complexities.

The advanced mathematics deployed in the biophysics of vision makes for difficult reading. There is a paucity of step-by-step reviews of this subject. Our approach is heuristic and at the end of this review, one should be able to follow superficially the algorithmic steps in understanding visual edge detection using Gabor filters. Therefore, we will adopt the Socratic method of asking questions and providing answers to help us through the complex web of mathematics.

In a nutshell, we will show that a Gabor filter is the inner product of a Gaussian distribution and the wave function. The Fourier transform of the convolution of the Gabor filter and the stimulus intensity function is what is recorded from simple visual cortical cells. This is not a coincidental observation, as nature economizes and utilizes a function that minimizes the uncertainty principle of signal extraction.

## Introduction and background

There are two basic models of early cortical visual processing attributed to Hubel et al. and Campbell et al. [1-2]. These papers are seminal and they are known as the Huber and Wiesel paper of 1962 and the Campbell and Robson paper of 1968. The key and relevant aspects of these papers are summarized in Table 1.

**Table 1:** The two main models of early visual cortical processing.

	HUBEL and WIESEL	CAMPBELL and ROBSON
MAIN IDEA	Simple cortical cells as feature detectors of bars and edges of various orientations and widths	Simple cells as Fourier analyzers of spatial frequency-tuned channels
DISADVANTAGES	Sketch too primal	Wide bandwidth, 1.6 octaves

These two models fall short of explaining the totality of the complexity of a visual scene, and they are referred to as primal models. A good model has to convolve all the features of a scene: contrast, texture, orientation, color, and reflectance. In addition, a good model has to convolve an image in space and time, integrating the fine-grain features with a cinematographic view so that an image is not degraded [3]. The ability to perceive motion is outside the scope of this review. To build up a theory of visual edge detection, we will begin with the concept of contrast sensitivity.

**Figure 1:**
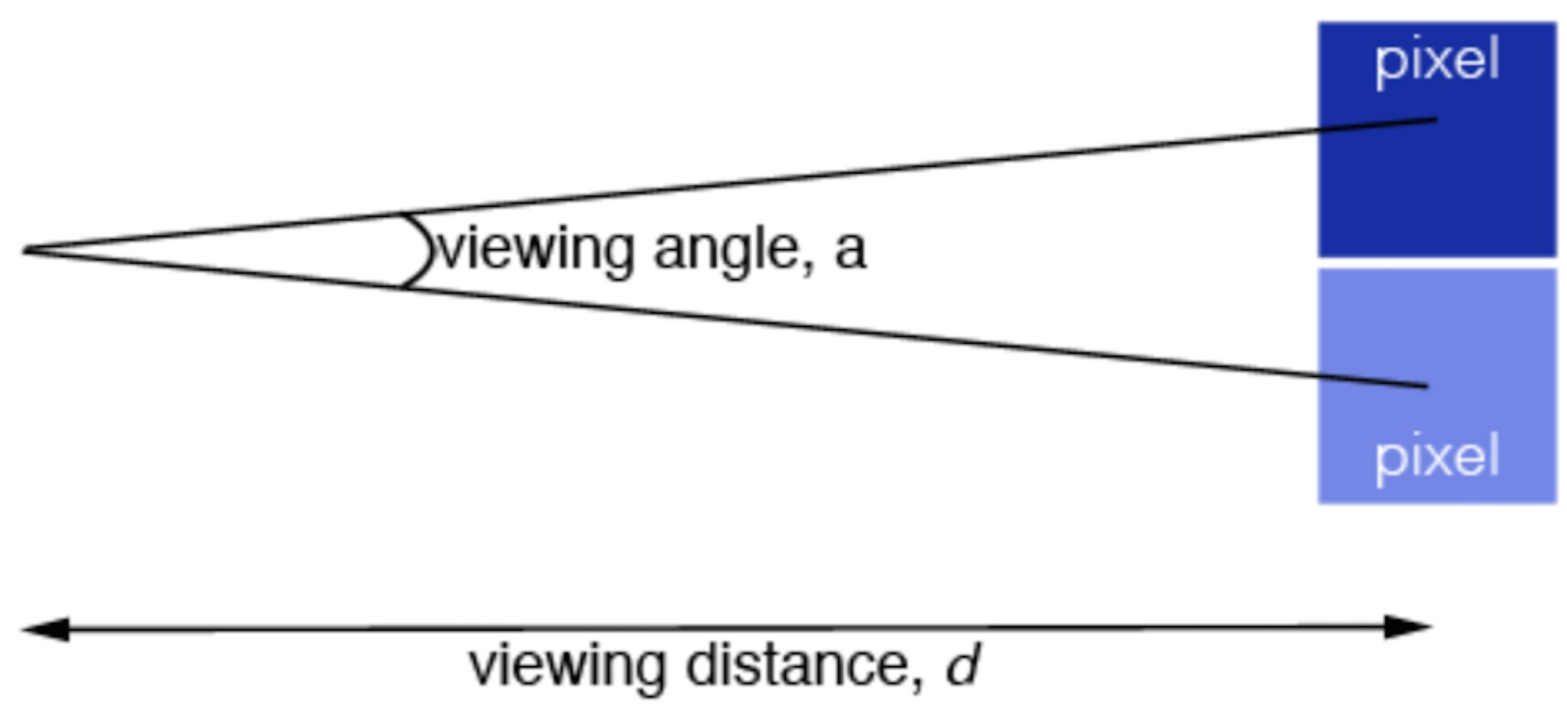
Concept of spatial frequency - distance (d) is the horizontal distance of the pixels from the eye, angle (a) of two pixels subtended by the eye

The clinical method of contrast visual testing (CVT) has offered deep insights into the dynamics of visual perception. CVT is a technique that involves contrast threshold testing of the visual system. This is akin to the ability to see a sharp outline, as in alternating bands of dark and white vertical bands. In order to understand CVT, we need to understand the concept of spatial frequency. Spatial frequency is a property that depends on the angle subtended by the receptive field as explained in Figure 1.

Spatial frequency depends on the distance ($d$) between pixels and the eye; the larger $d$ is, the smaller the separation of the pixels and vice versa. This means that contrast sensitivity decreases with the distance of an object from the eye, an intuitive relationship [4].

To cite an example, consider visual acuity testing with the Snellen chart. Here, the $d$ is fixed and we are limited to one spatial frequency. Visual acuity of 20/20 means that at a distance of 20 feet, a letter subtended at an angle of one-minute visual angle is visually discerned. No letter at a smaller visual angle than a one-minute degree can be discerned. Here, it is assumed that the visual contrast is at 100%. The Snellen chart does not allow us to determine the sensitivity of smaller objects with lower contrast. If the visual acuity is greater than 20/20, say 20/15, the threshold of vision detection decreases, which means that the sensitivity of the visual system increases.

**Figure 2:**
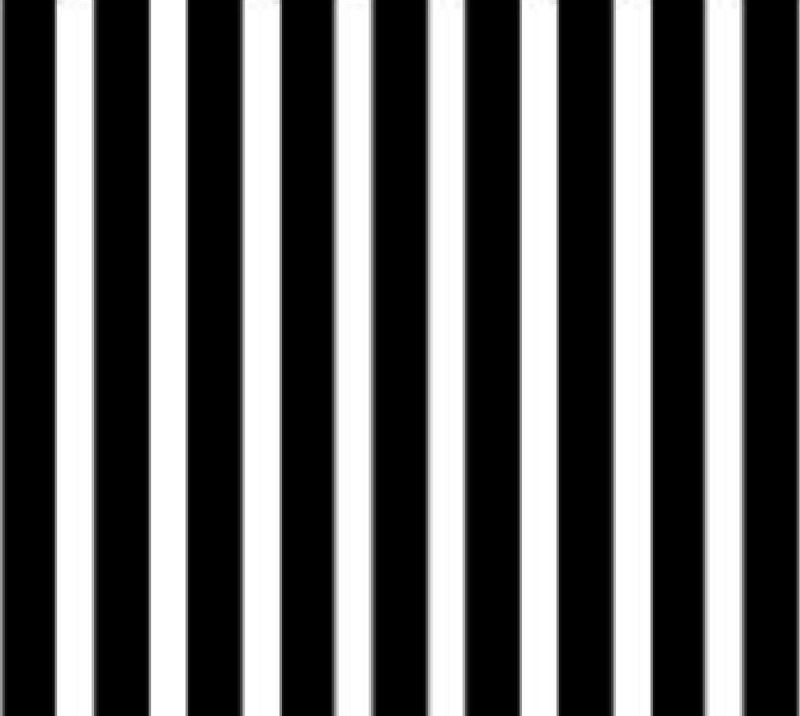
Example of a sine-wave grating

The one-minute of subtended angle means that the Snellen chart evaluates vision at one spatial frequency. The spatial frequency scale ranges from very coarse to very fine. The standard method to cover the whole bandwidth of vision is to use sine-wave gratings. These gratings are alternating white and dark bands that are exposed at a standard light level. We can then think of spatial frequency as the number of alternating white and dark bands subtended by one degree of angle. An example of a sine-wave grating is shown below (Figure 2).

At low enough contrast, the grating above will be hard to see. At higher contrast, the grating becomes more visible. The minimum contrast at which the alternating bars can be differentiated is the contrast threshold. Typically, five sine-wave grating frequencies with different contrast scales are deployed to cover the range of spatial frequencies, from 1.5 Hertz (Hz) to 18 Hz [5].

The gratings can display four features: the first is the number of alternating dark and white bands, the frequency, subtended at a fixed angle. That is, the number of white and dark bands spanning one degree of subtended visual angle. The contrast is the intensity of the alternating dark and light bands. The phase of the grating is the position of the grating with respect to a fixed background. The last feature is the orientation, which is the tilt of the grating.

**Figure 3:**
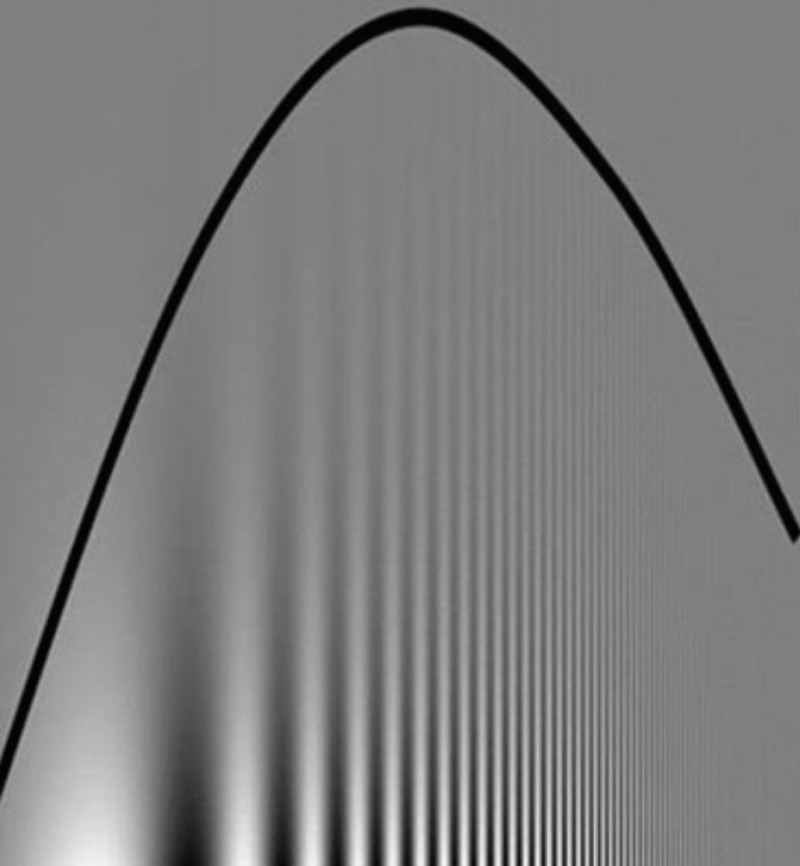
Bell-shaped curve of contrast sensitivity function; frequency on the abscissa and contrast sensitivity on the ordinate axis Note the increase in frequency of alternating bands as we move from left to right. The bell-shaped curve is superimposed on a background of a sine-wave grating that displays reduced contrast sensitivity as we go further up the diagram. Background grading is known as the Campbell-Robson figure.

Visual cortical cells are separately tuned to different spatial frequencies [2]. By altering the frequency of the alternating bands and the contrast or intensity of brightness, one can establish a contrast sensitivity (CS) curve, also known as a spatial frequency tuning curve. Physicists use the term Transfer Function. The information that can be gleaned from a CS curve is impressive (Figure 3).

There is a reciprocal relationship between contrast sensitivity and contrast threshold detection. Low threshold detection implies high sensitivity and vice versa. The contrast sensitivity curve is obtained by plotting the contrast sensitivity in the ordinate axis and frequency of the alternating bands in the abscissa. In this instance, for clarity, the curve is displayed against a background of varying frequencies and contrast of the grating. Note how the frequency varies from low to high frequency from left to right and note how the contrast threshold diminishes as we go further up the grating but the sensitivity increases, a reciprocal relationship. A bell-shaped curve is obtained.

Note the low contrast sensitivity at both low and high spatial frequencies. At mid-frequency, around 4 Hz, the visual contrast sensitivity is the highest. This bell-shaped curve of contrast sensitivity against spatial frequency is determined by an ensemble of neurons and is determined by the number of neurons and their firing rate at a specific spatial frequency [2].

The very last point of the curve, which is the highest resolvable frequency, coincides with the visual acuity, a powerful observation. Another feature of the CS curve is that various frequency bandwidths correspond to the different spatial frequency channels of the visual system. Furthermore, each frequency range corresponds to a different visual mechanism. The point at which the curve crosses the abscissa, the horizontal axis, is the cut-off frequency. At this point, the contrast is zero and the bands cannot be separated. When the descending limb of the bell-shaped curve is shifted to the left, the image is more degraded and smeared. This can occur with aging or with an immature visual system, as in infancy. High spatial frequency correlates with fine detail and low spatial frequency correlates with coarser details [2,4-5].

Sine-wave grating slides are standardized into five sets: A, B, C, D, E. They come in steps of one octave, except steps D and E, which are separated by one-half of an octave of contrast. The spatial frequencies are listed below. The contrast gratings change by 0.15 log units as we move from one set to another, either ascending by 100% gain or descending by 50% loss of contrast from step E to D (Table 2).

**Table 2:** Demonstrating the standardized sine-wave grating frequencies; noting the doubling of frequencies except the jump from grating D to E Hertz (Hz)

A	B	C	D	E
1.5 Hz	3 Hz	6 Hz	12 Hz	18 Hz

The five sine-wave sets cover the bandwidth of vision and are deployed to measure contrast threshold and, by default, contrast sensitivity. They can be displayed in tabular or graphic form, with the graphic form usually forming a bell-shaped curve. The display is computerized. Each eye is tested separately. The patient adjusts the knob for brightness in order to determine the threshold of detection, which is the luminosity at which the vertical alternating bands can be visually separated [6].

In summary, vision is perceived by the visual system integrating the relative size of an object, which is determined by the subtended angle and by the relative contrast of the object. The threshold contrast is reciprocally related to the contrast sensitivity. Contrast sensitivity is functionally related to the spatial frequencies, and the relationship is a bell-shaped curve.

CVT is also useful clinically. In patients with normal visual evoked potentials (VEP) and normal visual acuity of 20/20, CVT can detect subclinical visual dysfunction, as in the early papilledema of pseudotumor cerebri (PTC). Impaired CVT usually precedes prolonged VEP latencies, which is a later phenomenon in PTC. The former is thought to be due to impaired axoplasmic flow and the latter due to ischemia to the optic nerve. CVT was only abnormal in 17% of patients with drusen of the optic disc, pseudopapilledema, differentiating this entity from optic disc edema, papilledema. The loss of retinal ganglion cells renders all symptomatic patients with Leber’s hereditary optic nerve edema with an abnormal CVT. CVT was more sensitive than VEP in acute optic neuritis, being abnormal in 100 % of patients [5-6].

Simple cortical visual cells were described by Hubel and Wiesel [1]. They are tuned to a spatial dimension variable and to a spatial frequency variable. The spatial dimension variable is tuned to excitatory and inhibitory elongated visual perceptual fields, rendering them sensitive to edge or line detection. They are also tuned to spatial frequencies of one-octave bandwidth. The latter property is equivalent to the visual cortex acting as a spatial frequency analyzer. This hypothesis fits with the experimental observations of the spatial frequency tuning curves with contrast visual tests using gratings, as described above. Paraphrased mathematically, this means that the visual cortex computes a two-dimensional Fourier transformation of the percept, the object of visual perception [1].

The Gabor transform provides the mathematical machinery to carry out these computations at the earliest cortical level in the processing cascade [7-8]. Its main features are:

1. To convolve both the spatial variable and spatial frequency parameters simultaneously 

2. Allow the visual signal, which is expressed as an infinite Fourier series, to be reduced to a finite sum and guarantee convergence, by convolving it with a Gaussian distribution

3. To reduce a complex two-dimensional problem to a one-dimensional problem without loss of generality

4. Convolution of functions is a linear process, in keeping, with the linear summation of simple visual cortical cell function

A comparison of simple and complex visual cortical cells is provided in Table 3 [9].

**Table 3:** Comparison of the properties of simple and complex visual cortical cells Visual (V)

SIMPLE CELLS	COMPLEX CELLS
Layers 4 and 6, V1 cortex	Layers 2,3 and 5; V1, V2, and V3 cortex
Distinct excitatory and inhibitory regions within their receptive fields	No fragmentation of receptive field; spatial invariance
Summation is linear - additive	Orientation dependent - responds to edges
Cancellation effect when "on" and "off" zone activated simultaneously - mutual antagonism	Uniform stimulation of receptive field evokes no response- contrast necessary - responds to gratings
Responds to both oriented edges and gratings	Responds to both oriented edges and gratings
Gabor wavelet transform	

Visual cortical cells are excited by the percept, the object of perception. What does this mean? How does this titillation or stimulation lead to a visual construct? This is a deep philosophical conundrum. This question goes back to ancient antiquity and has challenged philosophers and, more recently, physiologists. Avicenna, the great 11th-century polymath who wrote the Canon of Medicine, a pretext for medicine until the Renaissance era, battled with the idea of essence versus existence and how we perceive the essence of a percept and its implication for existence [10].

The answer lies in the power of Fourier transforms and mathematical convolutions. As we explain later in more detail, physical variables in nature exist as a Fourier pair. To cite one example, the frequency of a sound, no matter how complex, can be conjugately paired with the time variable [11]:

$$f(t)=\frac{1}{2\pi }\int_{-\infty }^{\infty }F(\omega ).e^{i\omega t}d\omega$$

$$F(\omega )=\int_{-\infty }^{\infty }f(t).e^{-i\omega t}dt$$

where, $\omega$, is the frequency and, $t$, is the time.

The excitation of a visual cortical neuron has a Fourier pair, the stimulus intensity of a percept. The number of neurons in this ensemble is Fourier paired with a function, known as the filter function. The giant leap arises when we convolve these two pairs by an easy-to-prove mathematical theorem, the Convolution Theorem. By applying this theorem, if we convolve the number of neurons in an ensemble with the function of excitation of individual cells, its Fourier pair is the convolution of the stimulus intensity and the filter function [12].

This is a powerful statement. It means that the very act of selectively activating an ensemble of neurons leads to a visual construct. We will dedicate the second half of this review to unfolding the mathematical machinery in order to arrive at the mathematical construct of a Gabor transform and outline the impressive experimental data supporting this hypothesis. Due to the complexity of the mathematics, which involves advanced group theory, only a superficial heuristic approach is undertaken to highlight the concepts.

There are three preliminary ideas that we need to explore at the outset. The concept of convolutions, why the distribution of neuronal ensembles is a Gaussian distribution, and the uncertainty principle of signal analysis.

For simplicity, we will illustrate the concept of convolutions as it applies to probability theory [13]. Take two random variables, $r$, with probability, $P(r)$, and a variable, $s$, with probability, $Q(s)$. The question is; what is the probability of $r$ and $s$? To answer this question, we let $u = r + s$, then 

$$P(r+s) = P(r).Q(u-r)=P(r).Q(s)=P *S$$,

where $*$ is the convolution. 

The reason for deploying the Gaussian distribution with neuronal ensembles is that neurons send out dendrites by a random walk [14]. The probability that the $i$-th neuron is connected with the $j$-th neuron, is greater than the distance, $d_{ik}$, follows a Gaussian distribution, with standard deviation $\sigma$, and is proportional to the exponential

$$e^{\frac{-d_{ik}^{2}}{\sigma ^{2}}}$$

The uncertainty principle of signal analysis was initially outlined by Gabor, and we will re-iterate this idea further in the Review section. It states that there is a trade-off between the time-resolution and frequency-resolution of a signal or waveform. The product of the change in frequency times the change in time has a lower bound. There is a minimum area and the infimum of this area is optimized by a Gabor transform, which is a product of a Gaussian distribution and a complex exponential [15]. More on this to follow in the Review section.

We will state the Convolution Theorem of Fourier transforms, as we shall apply it frequently. It states that the Fourier transform of the product of two functions $\mathrm{f}$ and $\mathrm{g}$ is equal to the product of the Fourier transform of each function individually. Stated mathematically:

$$\mathfrak{F(\mathrm{f}*\mathrm{g})}=\mathfrak{F(\mathrm{f})}*\mathfrak{F}(\mathrm{g})$$

## Review

Psychophysics

Weber's Law and Logarithmic Functions

The CVT curve is a bell-shaped curve. This is not a coincidence. Perception is a logarithmic function and the mathematical integral of a bell-shaped curve is a sigmoid function, which is a logarithmic function. This is Weber’s law, a law of psychophysics, which is the science of sensation and perception [16].

E.H. Weber, a nineteenth-century physiologist, noted that a person could not discern the difference of 0.5 grams (g) of two weights placed on the palm, 20 g and 20.5 g, but could differentiate a 20 g weight from a 21 g weight. As the weight increases, say to 80 g, a person needed an increment of 5 g, to distinguish an 80 g from an 85 g weight. One cannot distinguish an 80 g weight from an 81 g weight or an 83 g weight. The increment has to be 5 %, equal to the percent difference between a 20 g weight and a 21 g weight. This was known as the just noticeable difference (jnd) [16]. This can be expressed mathematically as:

$$jnd=kS^{r}$$

where $k$ is a constant, $S$ is the stimulus intensity, and $r$ is a fraction

By logarithmic conversion,

$$log(jnd)= rlog(S) +B$$

where $B$ is a constant.

Perception is a logarithmic function and the curve of $jnd$ against the stimulus, $S$, is a sigmoid function. The derivative of a sigmoid curve is a bell-shaped curve. This property of perception is universal in the animal kingdom [16]. It also applies to vision, as we graphically demonstrated for CVT. A practical and simple example of Weber's law is musical scales; an octave corresponds to the frequency ratio of 2:1, a fifth to a ratio of 3:2, a fourth to a ratio of 4:3, and so on. To cite an example; the frequency increases in a geometrical pattern of 1, 2, 4, 8, 16, ....; noting an exponent of 2, when the notes are separated by an octave. This is a logarithmic scale.

Fourier analysis of a square-wave

Any waveform, simple, complex, or discontinuous can be expressed as an infinite sum of sine and cosine waveforms, a series, S(x) [17], (Appendix $\mathrm{I}$). Let us examine an infinite series of sine waves.

**Figure 4:**
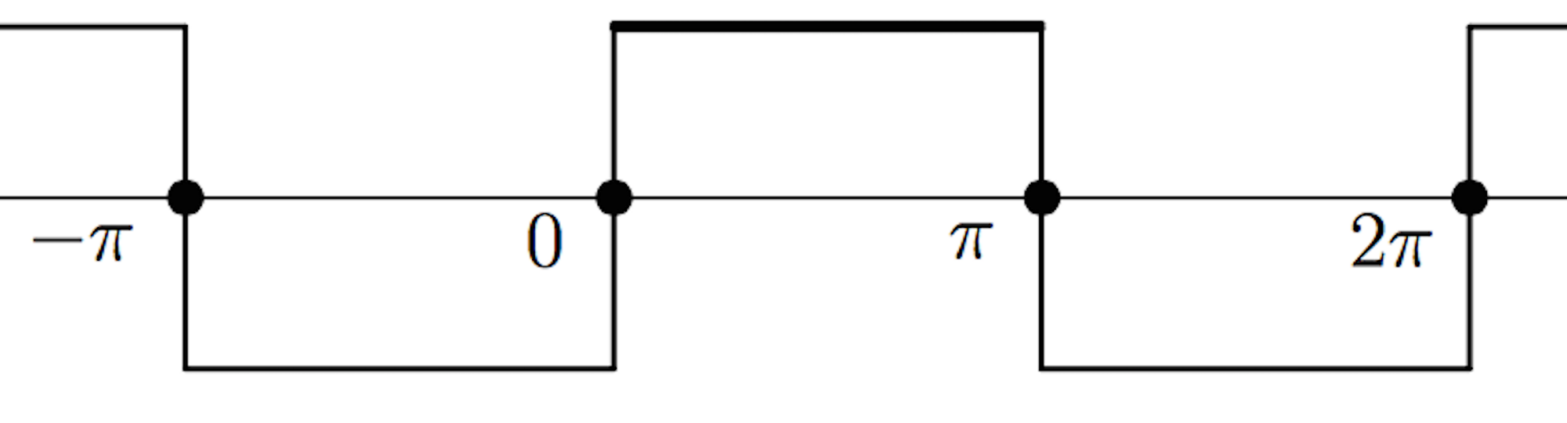
A square-wave function

We next apply the Fourier method to a square-wave function, SW(x) = 1, $0 &lt; x &lt; \pi$, (Figure 4).

We apply the Fourier coefficient integral for $S(x)$= 1, $[0,\pi ]$

$$b_{k} = \frac{2}{\pi }\int_{0}^{\pi } 1.sin(kx) dx = \frac{2}{\pi }[\frac{2}{1},0,\frac{2}{3},0,\frac{2}{5},0,...] = \frac{4}{k\pi }$$

 where k is a positive odd integer

Therefore, the square wave function, S(x), can be expressed as:

$$S(x) = \frac{4}{\pi }[\frac{sin(x)}{1}+\frac{sin(3x)}{3}+\frac{sin(5x)}{5}+ ......]$$

**Figure 5:**
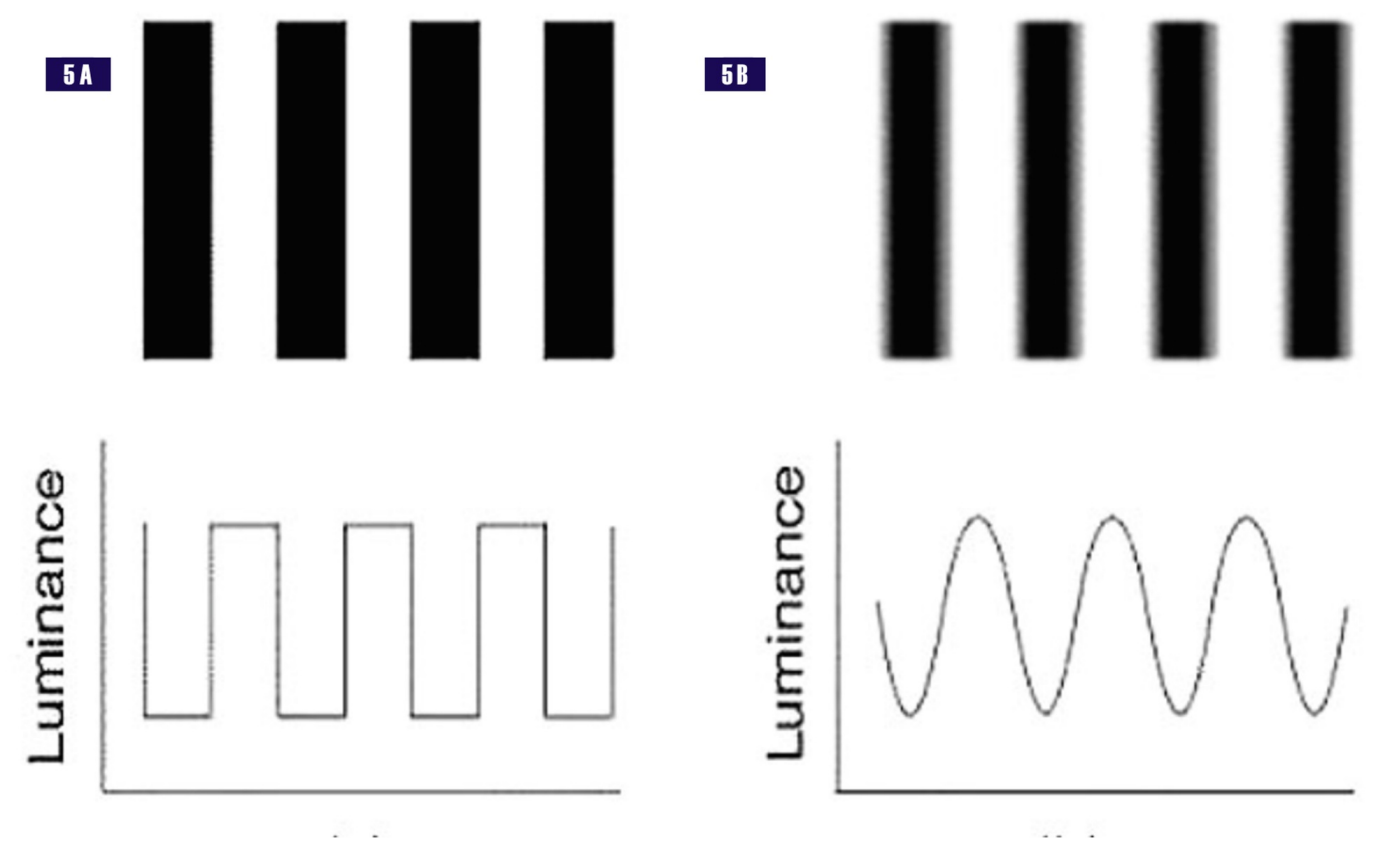
Examples of gratings. 5A - rectangular grating. 5B - sine-wave grating Note how luminance fades in dark bars of sine-wave gratings as compared to the rectangular grating.

The gratings can have a sine, square, rectangular, or saw-tooth profile. Listed are examples of a rectangular and a sine-wave grating (Figure 5).

The Fourier series can be computed for different functions $S(x)$, as we demonstrated for the square-wave grating above. The amplitude of the contrast threshold is determined by the coefficient of each term, or harmonic, of the Fourier series. For example, for the square-wave function, if the contrast is, $m$, the amplitude of each harmonic is:

$$\frac{4m}{\pi }, \frac{4m}{3\pi }, \frac{4m}{5\pi }, ......$$

noting that the even harmonics are of zero amplitude, as one can see from the Fourier series. Listed are the first, third, fifth, and, by extension, the higher harmonics. Campbell et al. demonstrated that the square wave and other more complex gratings could not be distinguished from the sine-wave gratings until the higher harmonics of their Fourier components could be detected at their contrast threshold [2]. This was explained by the visual system consisting of different channels selecting a specific bandwidth. Paraphrasing, different visual channels have their own spatial frequencies.

Enroth-Cugell et al., showed experimentally in cats, that this spatial frequency selectivity, or band-pass filtering of contrast sensitivity, or selective tuning begins at the level of the retinal ganglion cells [18].

Wavelet theory

The Fourier transform switches from the frequency domain to the time domain and vice versa. Fourier transform provides frequency resolution without time resolution. We can derive spectra of frequencies without any information on the timing. The question is: how can we display time and frequency data in the same domain? The answer is a short duration signal known as a wavelet and its transform. We compare a Fourier transform with a wavelet transform (Table 4).

**Table 4:** Comparison of Fourier transforms and wavelet transforms

FOURIER TRANSFORM	WAVELET TRANSFORM
Frequency information only	Frequency and time information
Does not work with a burst of data or discontinuous signal	Works with short signal and edge detection
Analyzes the whole signal only - lose frequency of short-duration segment	Does not lose the short-duration signal
Power spectra	Scale and shift signal-scale gives frequency band - calculate correlation coefficient
Only two base functions - sine and cosine	An infinite number of basis functions

**Figure 6:**
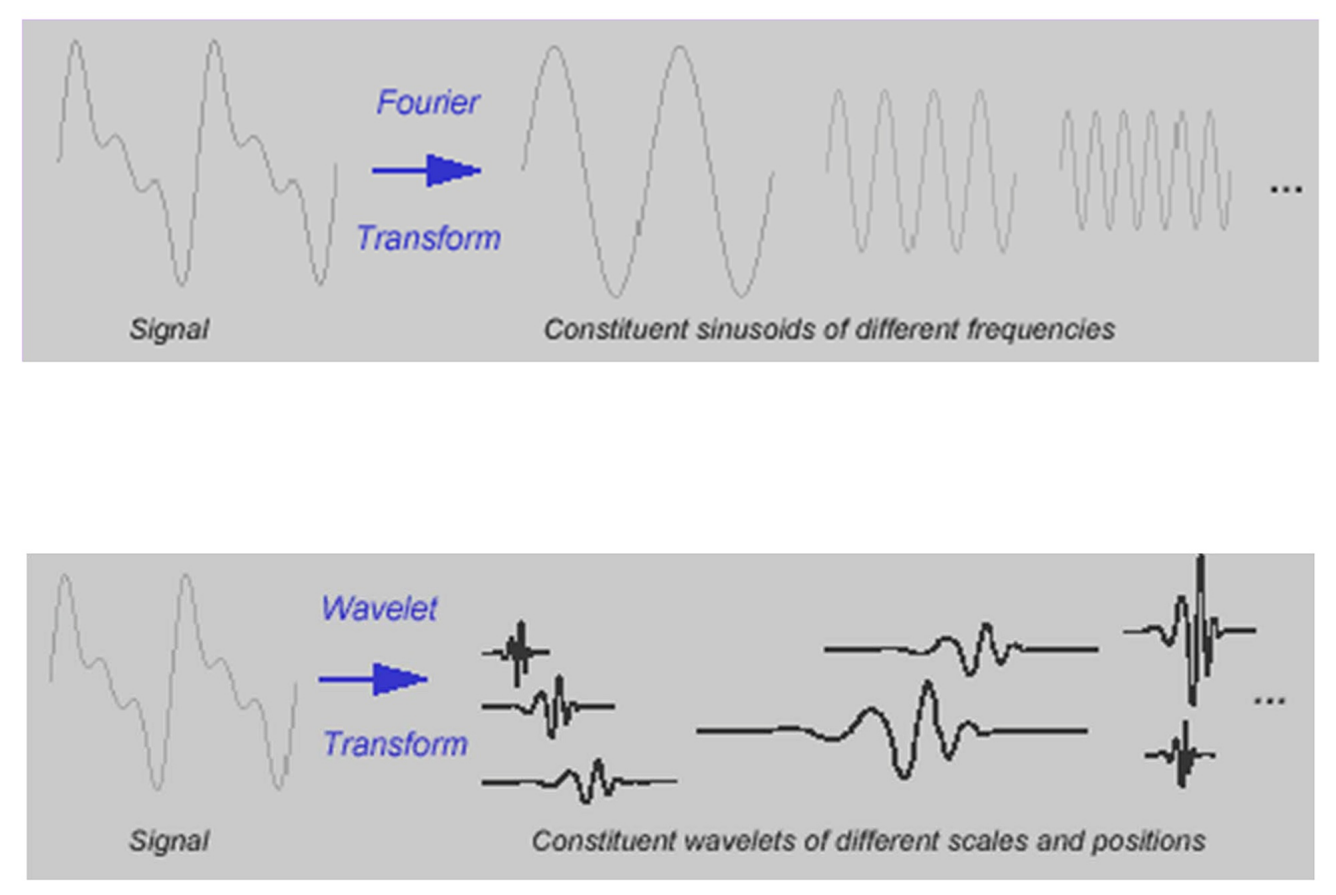
Comparing a Fourier transform with a wavelet transform

As the table demonstrates, the wavelet transform is versatile, allowing the simultaneous measurement of the frequency and timing of a signal. Its flexibility allows it to compute edges and discontinuities and filter out high-frequency noise. We introduce the idea of a "windowed Fourier transform," which cuts a signal into sections and each section is analyzed separately. A longer time interval will capture low-frequency fluctuations and a short time window will capture high-frequency fluctuations. The basis functions from which all signals are constructed is infinite. A wavelet, by definition, is a waveform of very short duration with an average value of zero [19-20]. The decomposition of a signal by Fourier transformation and wavelet transformation is graphically demonstrated for comparison (Figure 6).

**Figure 7:**
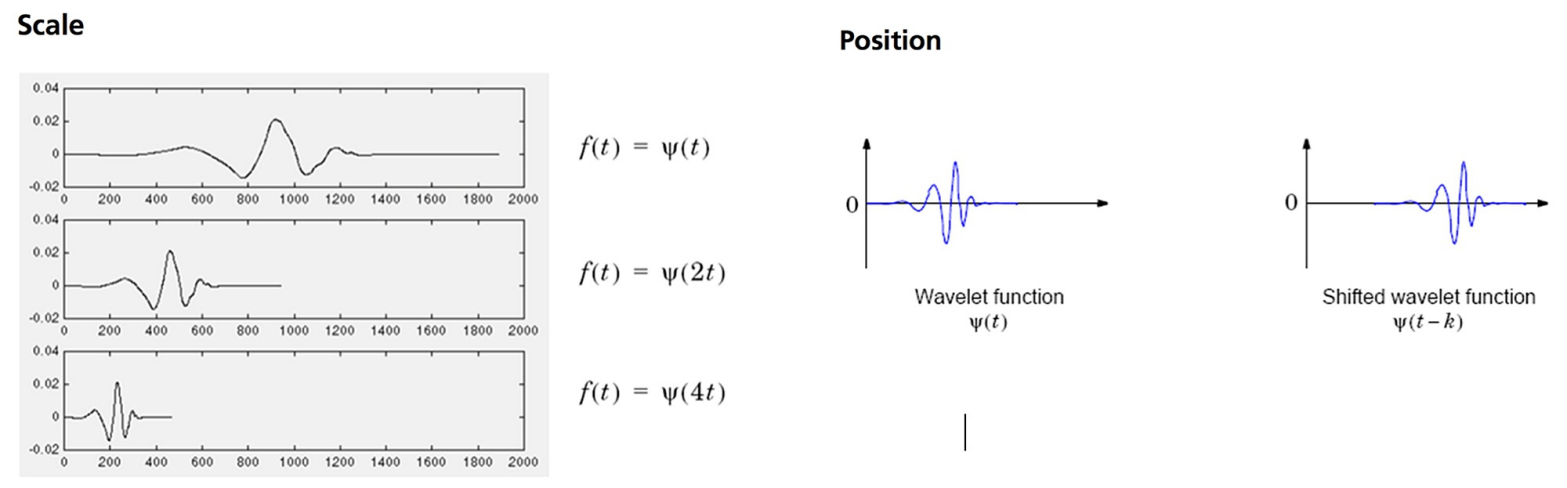
Demonstration of scaling and position parameters of wavelets

The scale is the process of compressing or spreading the signal and the position is the shift of the signal along the horizontal axis (Figure 7).

**Figure 8:**
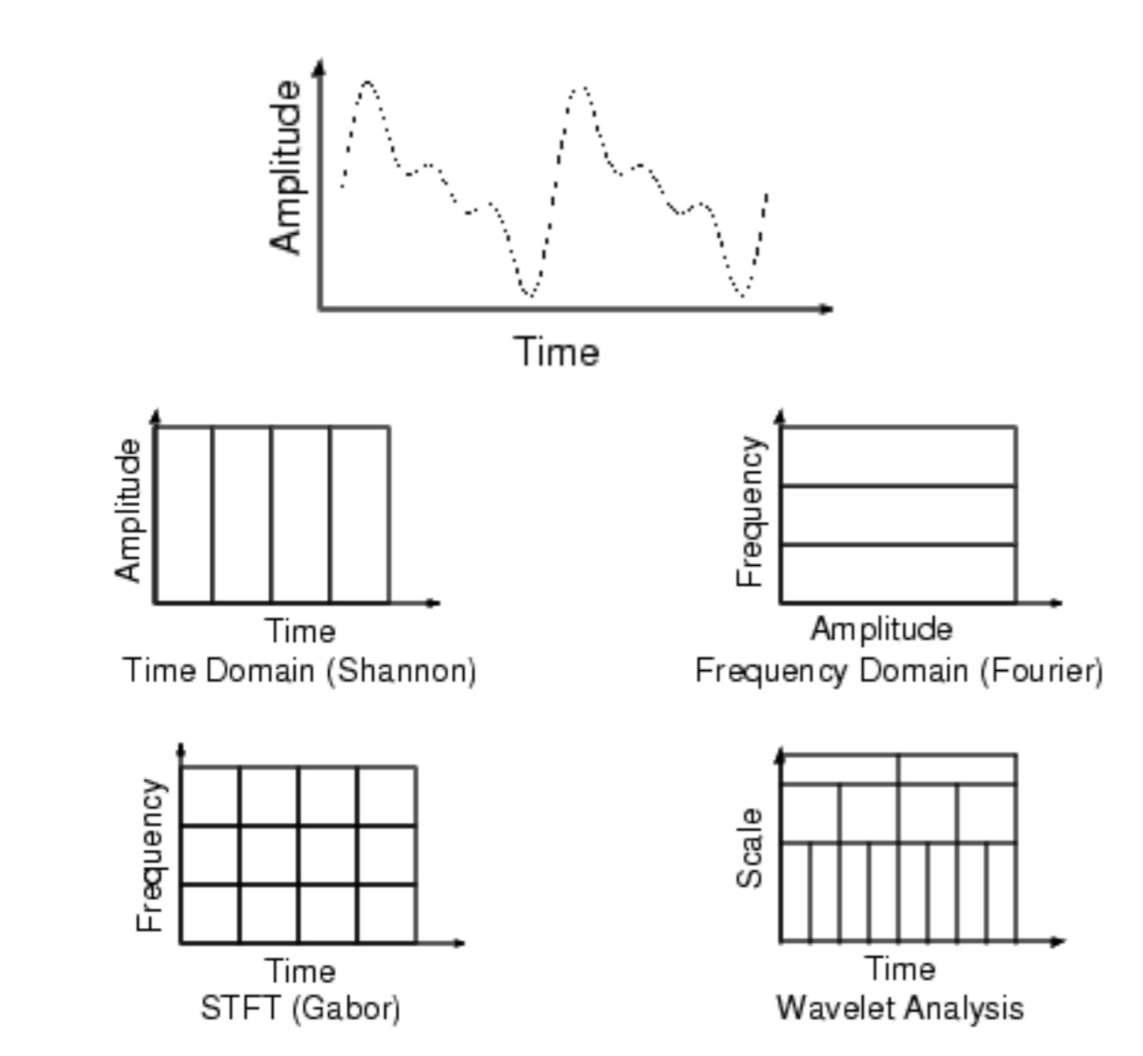
Comparison of various transformations Short-time Fourier transform (STFT)

Comparing various transformation methods reveals that wavelet analysis provides fine and coarse grain resolution unparalleled by the other methods (Figure 8).

In summary, with a small scalar value, a wavelet is compressed and rapidly changing details can be extracted as a high-frequency signal; fine-graining. In converse, with a large scalar value, a wavelet is stretched, and slowly changing low-frequency details are extracted; coarse-graining.

Mathematically, we can express the continuous waveform transform as the sum of the overtime of the signal multiplied by the scaled and shifted versions of the wavelet function, $\Psi (t)$. If $f(t)$ is our original signal, $s$ the scale factor, and $x$, the position, our wavelet coefficients are $F(s,x,t)$ is:

$$F(s,x,t)=\int_{-\infty }^{\infty }\Psi (s,x,t).f(t)dt$$

This is how we decompose a signal into its basis wavelets. We can also think of it as the dot product between the wavelet function and our signal function.

There is a single " mother wavelet " form in which all wavelets are derived by scaling, $s$, and translation, $x$

$$\Psi _{s,x }^{*}(t)=\frac{1}{\sqrt{s}}.\Psi (\frac{t-x }{s})$$

**Figure 9:**
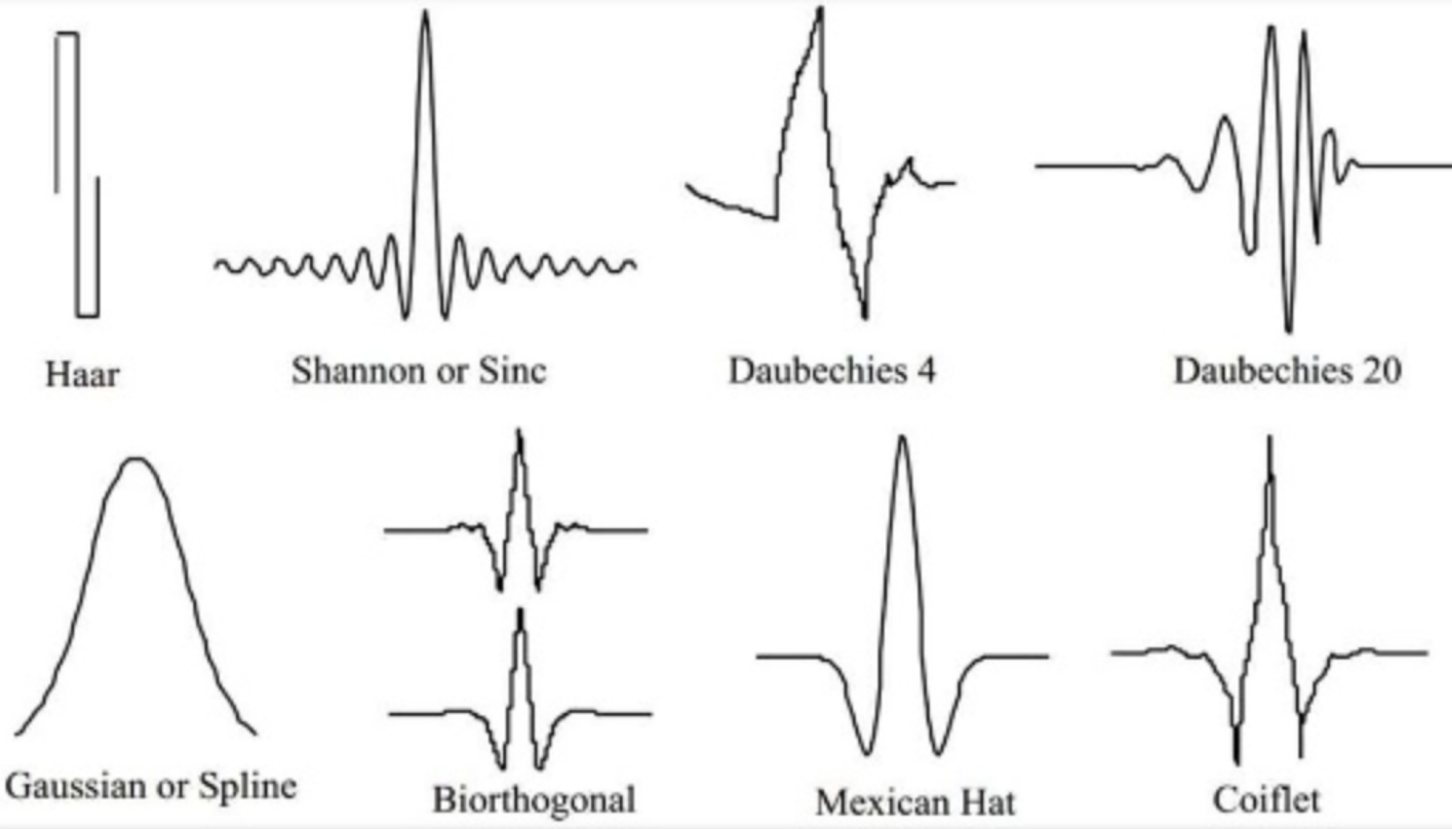
Different types of mother wavelets

There are different mother wavelets (Figure 9).

For our purpose of visual edge detection, we are interested in the Mexican hat mother wavelet, which we will derive heuristically.

The meaning of perception

Definitions

The Fourier transform is the tool we deploy in dealing with spatial frequencies [12]. The visual cortex, V1, analyzes visual information by computing the signal intensity of an image, say, $S(x)$, on the retina. To simplify the mathematical equations, we will consider the one-dimensional case. The idea is the same for higher dimensions. We want to calculate the magnitude of the receptive field function, a planar complex waveform, $\Psi (\nu$,x)\) in the visual cortex. A certain population of neurons reacts to a certain region of the visual object, our percept. The complex plane wave is expressed at a location, $\nu$, in the visual cortex, at a location, $x$, in the retina. The response, $\Psi(\nu ,x)$, is known as a receptive field function and is expressed mathematically as:

$$\Psi(\nu ,x)=e^{i\nu x}$$

The firing rate of a neuron, $F(\nu )$, is the inner product of the receptive field function of the neuron $\Psi (\nu ,x)$ and the magnitude of the stimulus, $S(x)$, at a position, $x$, on the retina

$$F(\nu ) =\int_{-\infty }^{\infty }dx. e^{-i\nu x}.S(x)= &lt;\Psi (\nu ,x),S(x)>$$

This is exactly the Fourier transform of the signal intensity of an image $S(x))$. We can also think of it as the dot product of the signal function, $S(x)$, and the receptive field function, $\Psi (\nu ,x)$.

Perception involves assigning a sub-population of neurons, $G(\nu )$, stacked at a point in the visual cortex, $\nu$, to a specific spatial frequency or a bandwidth of frequencies. This is known as a filter, $K(x)$. Note that $K$ depends on $x$, the activated locality in the retina [12]. This filtering function is how the perception of the image is constructed, a percept. The response of the sub-population of neurons, $R(\nu )$, is a product of the number of neurons, $G(\nu )$, multiplied by the Fourier transform, $F(\nu )$, of the receptive field of a single cell, $\Psi (\nu ,x)$.

$$R(\nu ) = G(\nu ).F(\nu )$$

The Percept as a Fourier Pair

In nature, physical variables come in conjugate or Fourier pairs. For example, in acoustics, the frequency, $f$, and time, $t$, come as a conjugate pair. In quantum mechanics, the position $x$ and the momentum $p$ come in conjugate pairs. Vision is no exception [13].

The firing rate of an ensemble of neurons, $F(\nu )$, at position, $\nu$, and the light intensity of an object at the retina, $S(x)$ come as a Fourier pair. The number of neurons in an ensemble, $G(\nu )$, and the filter function, $K(x)$, come as a Fourier pair. These are sublime and intriguing statements. This allows us to apply the convolution theorem and make the powerful statement that the convolution of the firing rate and number function of an ensemble of neurons is equivalent to the Fourier transform of the convolution of the filter function and light intensity of the object at the retina. By this computation, we have the machinery for constructing a percept [12,13]. This is expressed as:

$$G(\nu )F(\nu ) \leftrightharpoons K(\nu )S(\nu)$$

Gamma distributions and spatial frequency neuronal ensembles

Gamma Distributions

For $\alpha > 0$, the gamma distribution, $\Gamma (\alpha )$ is defined as:

$$\Gamma (\alpha ) = \int_{0}^{\infty }t^{\alpha -1}e^{-t}dt$$

The integrand $t^{\alpha -1}e^{-t}$ is positive for $t>0$; the probability density function, $pdf$, is defined as:

$$pdf = \frac{1}{\Gamma (\alpha )}t^{\alpha }e^{-t}, t>0$$

$\alpha$ is known as the shape parameter and the curve becomes less skewed as $\alpha$ increases [20]. To add a second parameter, we apply the transformation $y=\frac{t}{\beta }$ and we obtain:

$$pdf = \frac{1}{\Gamma (\alpha )}\beta ^{\alpha }y^{\alpha -1}e^{-\beta y}, y>0$$

$\beta$ is known as the rate parameter, which is the inverse of the scale parameter and it determines the shape of the distribution.

A useful property of gamma functions that is easy to derive is: 

$$\Gamma (\alpha ) = (\alpha -1)!$$

If we define $\omega$ as our spatial frequency, which replaces $y$, and $k$, the order of our distribution to replace $t$ and $a(E)$ to replace $\beta$, the rate parameter, we obtain:

$$pdf = G(a(E),k) = \frac{1}{(k-1)!}a^{k}(E)\omega ^{k-1}e^{-a(E)\omega }$$.

$a(E)$, the scale parameter, depends on the distance from the center of vision, the eccentricity.

**Figure 10:**
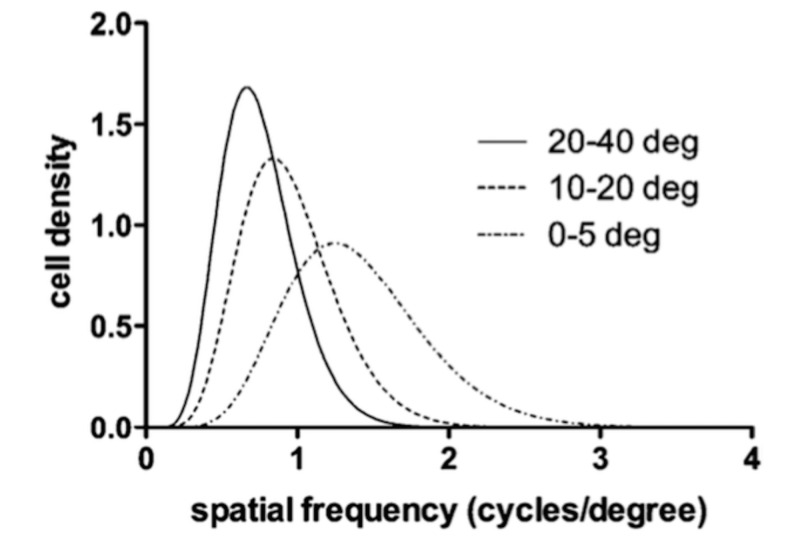
Spatial frequency tuning curves - different neuronal ensembles are tuned to different spatial frequencies providing a framework for the filtering function deg (degree)

A remarkable finding is the fact that some spatial frequencies have more cells devoted to them in their neuronal ensembles than other spatial frequencies and the distribution of these cells across various eccentricities follows a gamma probability density function of order $k=9$. These findings are in agreement with the Campbell-Robson plot of contrast sensitivity against spatial frequencies [21-22], (Figure 10).

The concept of edge detection

In analyzing an image, there are several parameters that need to be tuned: the contrast of the object and its shading, distance, and orientation. The contrast usually occurs over a wide frequency range. There is a trade-off between spatial and frequency resolution. This is the uncertainty principle of signal processing, which is expressed as:

$$\delta x.\delta \omega \geq \frac{\pi }{4}$$

The Gabor transform and the Gaussian distribution whose Fourier transform is a Gaussian distribution optimize the uncertainty principle [8,15].

If $x$ is the displacement of the plane wave, the Gaussian distribution is the usual formula:

$$G(x)=\frac{1}{\sigma (2\pi )^{\frac{1}{2}}}e^{\frac{-x^{2}}{2\sigma ^{2}}}$$

and its Fourier transform is: 

$$\mathfrak{F}(G(x))=e^{\frac{-\sigma ^{2}\omega ^{2}}{2}}$$

How do we define an intensity change?

By taking the second derivative of the convolution of the stimulus intensity $S(x,y)$ and the function of the group of neurons G(x,y) selectively activated by a certain part of the object and setting it to zero [23]. This is the zero-crossing of the curve.

$$f(x,y))=D^{2}[G(x,y)*S(x,y)]$$

By the derivative rule of convolutions:

$$f(x,y)=D^{2}(G).S(x,y)$$

**Figure 11:**
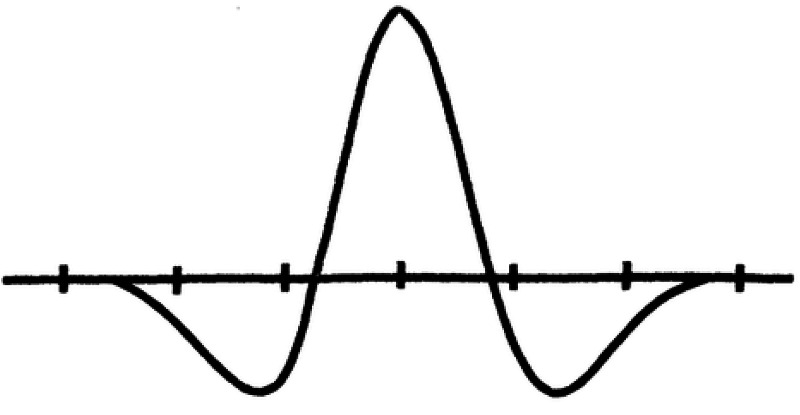
Mexican hat function - the second derivative of the Gaussian distribution of the activated ensemble of neurons

This represents the signature of an edge detector. It is also known as the Laplacian of the Gaussian (LOG). The graph of $D^{2}G$ looks like a Mexican hat and the zero crossings are where the graph meets the ordinate axis (Figure 11).

The zero-crossings are the regions of the image with the maximum change of intensity, that is, the boundary, edge, or contour.

Wavelet coherence and the Weyl group representation theory

How is that a perceived object is not distorted, magnified, contorted, or simply out of shape? Why is that a basketball is perceived as a spherical object? Why does the Mona Lisa have the appropriate disposition of facial features? The answers to these questions are found in the mathematics of symmetry groups, in group theory, specifically Lie groups. These groups carry the operations of translations, rotations, and dilations, and the group representations are outside the scope of this review. We will briefly sketch the basic ideas.

Firstly, we need to understand the concept of a coherent state, which has its origins in quantum mechanics. These are a set of states that exist in a Hilbert space, which is a finite or countably infinite, complex field. The Hilbert space is a vector space; in our case, an infinite-dimensional space, the $L^{2}$ space. We define this space as an inner product with respect to a measure, $\mu$, such that the square-integrable functions $f,g$ satisfy:

$$=\int_{X}^{}fgd\mu$$

where $X$ is a measure space, where the functions in $L^{2}$ satisfy:

$$&lt;\Phi /\Psi >=\int \Psi ^{*}\Phi dX$$

We will adopt the Dirac bra-ket notation of quantum mechanics [24]. Coherence is characterized by two properties:

1. The set of vectors $\left \{ |\Psi > :\Psi \in \mathfrak{H}\right \}$ form a continuous sub-manifold in the Hilbert space, $\mathfrak{H}$, which means that they are rarely orthogonal.

2. There exists a resolution of unity $I = \int |\Psi >&lt;\Psi |\delta \Psi$ over $\mathfrak{H}$, where the vectors are all of a unit length and a continuous change of phase leads to an equivalent set of coherent states.

Wavelets are functions generated from a "mother" wavelet by dilations and translations. The mother wavelet has localizable properties in the frequency and time domain. These wavelets are coherent states and the language that expresses them is the language of group representation theory. The phase space of these groups is the time-frequency domain. Gabor wavelets are a subgroup of the canonical coherent states related to the Weyl group. In one-dimension, the Gabor wavelets are generated by translation operations in an affine group and in two dimensions by the similitude group [25]. The similitude group, $SIM$, has a group representation:

$$SIM (2)= R^{2}\times (R^{+}\times SO(2))$$

where $R$ is the set of all real numbers and $SO(2))$ is the special orthogonal group, which is a rotational group. The first product in the $SIM(2)$ group representation is a semi-direct product. The $SIM (2)$ group is what is known as the Lie group. Lie groups are smooth maps, continuous groups that are a perfect model for continuous symmetry. Lie groups are ideal for preserving the geometry of a perceived object and preserving its shape as required by the visual system. For the definition of a mathematical group and Lie group, see Appendix $\mathrm{II}$. Simply stated, a Lie group is the group of continuous symmetry transformations. Think of a simple circle; we can rotate the circle infinitesimally and each infinitesimal rotation is symmetry. If we take an element $g$ close to the identity $I$, then:

$$g= I +\varepsilon X$$

where $\varepsilon$ is a very small number and $X$ is the generator of the group.

If we apply this infinitesimal transformation multiple times, we obtain:

$$h=(I+\varepsilon X)(I+\varepsilon X)(I+\varepsilon X)....=(I+\varepsilon X)^{k}$$

If we think of $\lambda$ as a finite transformation parameter and $N$ a very large number, we get:

$$g=lim_{N\rightarrow \infty }(I+\frac{\lambda X}{N})^{N} = e^{\lambda X}$$

 

Gabor transforms and filter function

The V1 visual cortex preserves the properties and features of objects as the image cascades proximally from the retina. Mathematically, this is expressed as a symmetry group with the elements of translation, rotation, and dilation, as noted above. In the jargon of this invariance group, there is a "mother wavelet" from which every possible translation, rotation, and dilation is derived [26-27].

The Gabor transform is the dot product of a Gaussian distribution with a complex exponential, the complex plane wave known as the receptive field potential. It consists of odd and even functions. The Gaussian distribution limits the spread of the complex exponential function and localizes it. We begin with a "mother wavelet," where the Gaussian function is known as the kernel. The expression is:

$$\Psi (x) = e^{\frac{-x^{2}-y^{2}}{\sigma _{x}^2{+\sigma _{y}^{2}}}}.e^{2\pi i\omega x} = e^{\frac{-x^{2}-y^{2}}{\sigma _{x}^2{+\sigma _{y}^{2}}}}.(cos(2\pi \omega x) + isin(2\pi \omega x))\frac{}{}$$

where $\omega$ is the center, the frequency at which the filter yields the greatest response, and $\sigma$ is the spread of the Gaussian window. We then translate the mother wavelet in the $x$-direction by a displacement of $r$, and in the $y$-direction, by a displacement of $s$. The group of translations is the affine group. For simplicity and without loss of generality, we will ignore rotations. I will perform all calculations for the mother wavelet. To generate other wavelets, we simply substitute $y-r$ for $y$ and $x-s$ for $x$ [12,28]. This makes the equations look cleaner for the beginner.

Note also that when $\omega =0$, we are left with a pure Gaussian curve.

**Figure 12:**
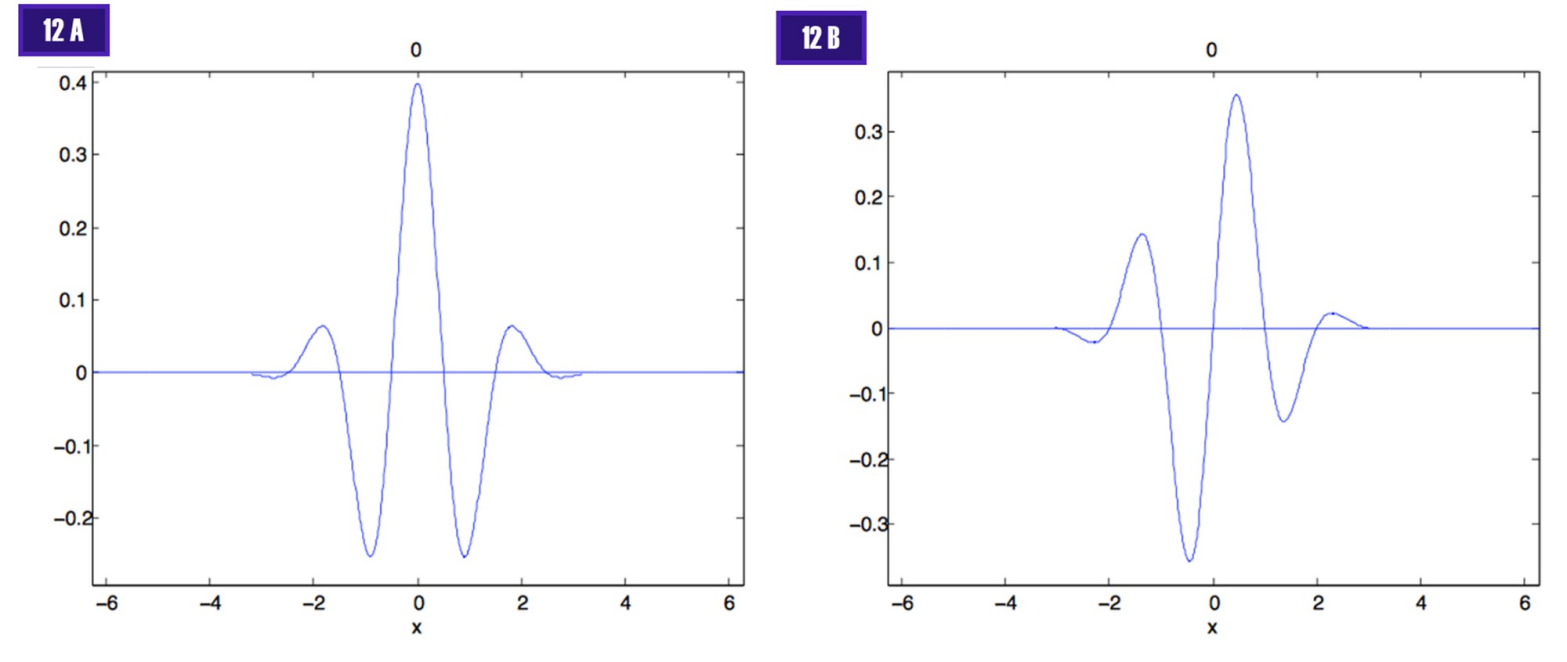
Even and odd Gabor transforms 12 A. Even - symmetric function. 12 B. Odd - anti-symmetric function

The graph of the even and odd functions are illustrated in Figure 12 [12].

The firing rate of a simple cortical cell is the inner product of $S(x)$ and the Gabor function [12]:

$$F(w)= \int_{-\infty }^{\infty }dx\int_{-\infty }^{\infty }dyS(x,y)\Psi ^{*}(x,y,\omega )=&lt;\Psi (x,y,\omega ),S(x,y)>$$

The next step is to complexify the firing rate $F(\omega )$ by multiplying it by $i\omega$. This step converts the firing rate into a wavelet transform. We define:

$$W (S(x,y))=i\omega F(x,y,\omega )$$

Why complexify the firing function? This concept lies at the heart of theoretical physics. It is the concept of Unitarity. Unitary operators preserve distances under the coordinate transformation (Appendix $\mathrm{III}$). A simplified idea is when we consider the complex number $i$, its complex conjugate is $-i$, and note that:

$$-i \times i = 1$$

returns the identity, $1$. In Appendix $\mathrm{III}$, we demonstrate a simple example of unitarity.

By applying the convolution theorem, we can convolve the visual scene with a spatial function as we pointed out in the introduction. Therefore:

$$G(\omega )F(\omega )=W[K(x)^{*}S(x,y)]$$

We can think of the filter function, $K(\omega )$, as the selection of an ensemble of neurons by summing a number of neurons that are dedicated to a specific spatial frequency $\omega$ and is defined as:

$$K(\omega ) =\int_{-\infty }^{x}duG(u)$$

The next step is to determine how this filter function by an ensemble of neurons leads to "edge detection" [12]. Let us begin with the response $R(\omega )$ of a group of neurons. As before, this is a product of the firing rate $F(\omega )$ and the dedicated ensemble and is a simple product:

$$R(\omega )= \mathfrak{F(\mathrm{F(\omega )S(\omega )})}$$

where $\mathfrak{F()}$ is the Fourier transform; we substitute for $F(\omega ) , S(\omega )$ and obtain:

$$R(\omega )=\int_{-\infty }^{\infty }dx.e^{\frac{-x^{2}-y^{2}}{2\sigma _{x}^{2}+2\sigma _{y}^{2}}}.e^{-i\omega x}\int_{-\infty }^{\infty }du.K(x-u).S(u)$$

Substituting $x=t-u$, we obtain, with re-arranging,

$R(\omega ) = \int_{-\infty }^{\infty }dt.K(t).e^{-i\omega t}\int_{-\infty }^{\infty }du.S(u).e^{\frac{-x^{2}-y^{2}}{2\sigma_{x}^{2}+2\sigma _{y}^{2}}}.e^{i\omega x} = \mathfrak{F}(K(\omega )S(\omega ))$

**Figure 13:**
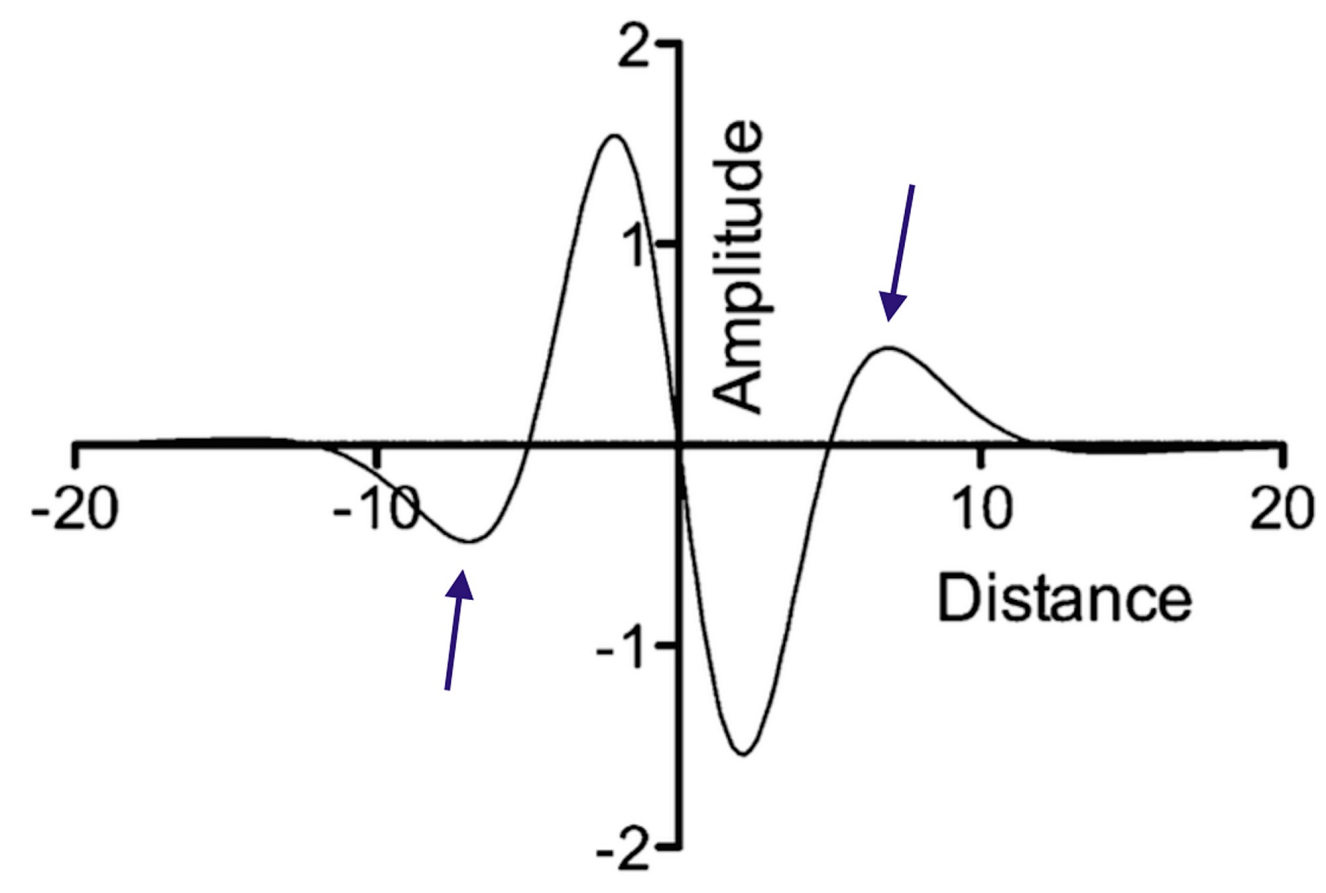
Filter function showing the characteristic edge detector graphic for 20-40 degree eccentricity with the Mach bands (blue arrows)

A graph of the filter function $K(x)$ against $x$, the measure of visual angle, fits with the signature of an edge detector. This plot is for 20-40 degree eccentricity (Figure 13) [12].

The Mach bands are an illusion due to lateral inhibition between adjacent bands of different contrast. This effect is enhanced when two bands are immediately adjacent to each other [29].

To summarize, a visual percept is constructed by a pattern of excitation of an ensemble of neurons. This is obtained by convolving the stimulus function of an object with the receptive field of the corresponding neurons. The receptive field is a plane wave due to neuronal excitation. The convolution of a plane wave with the Gaussian distribution is the Gabor transform. The Gabor transforms form an affine group with affine displacements. They form a Weyl group with rotations, translations, and dilations.

**Figure 14:**
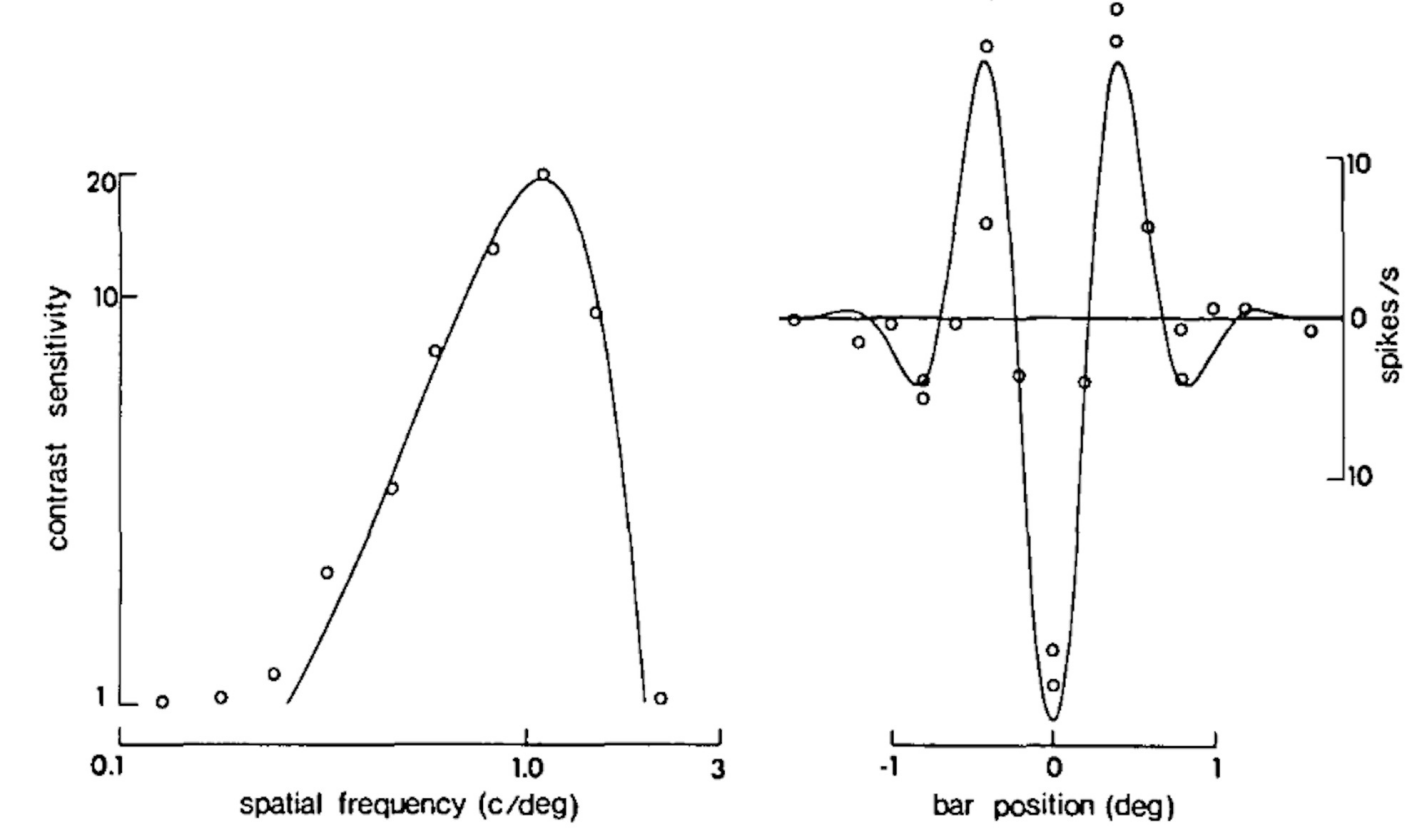
De Valois et al. recordings from visual cortical cells On the left is the typical frequency tuning curve; to the right is the even function Gabor elementary signal cycles per degree (c/deg), cycle (c), second (s)

The elementary Gabor signals have an odd (anti-symmetric) and an even (symmetric) component, which are found experimentally with simple cell recordings [30-31]. Different cortical cells respond to different spatial frequencies (Figure 14).

The synapto-dendrodendritic web

Is there a cortical anatomic substrate where these wavelets are formed and interact? The answer is in the affirmative. The study of these receptive electric fields is known as holonomy, and the plane waves carry out their action in the phase space, the Hilbert space. We know that the distal ends of axons split into teledendrons and form a web of interconnected fibers. These dendrites communicate through electrical and chemical synapses. Electric recordings reveal oscillations of depolarizations and hyperpolarizations of electric potential differences without electric currents. These oscillations in different cortical slices intersect with one another, producing waves of interference. Different neuronal ensembles overlap in this assembly of interfering complex plane waves, in a holographic manner [32-33].

Summary of findings

**Figure 15:**
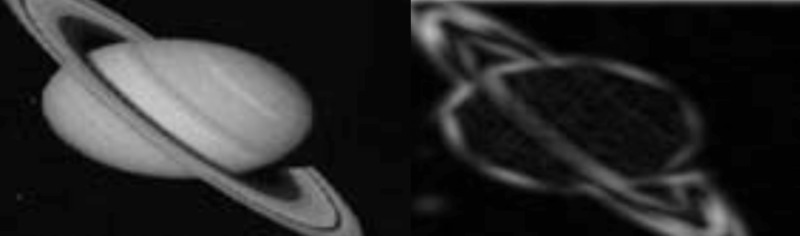
Gabor filter of the planet Saturn The right image demonstrates the edge effect of convolving the Gaussian function with the stimulus function.

Visual analysis involves feature extraction, with edge detection being a fundamental process. Edge detection involves analyzing discontinuities in an image and a change in contrast to the background. From edges or boundaries, other features, such as the perimeter, the area, and the contour or shape of an object, can be defined. Change means mathematical differentiation and involves first and second-order derivatives. We demonstrated how the Laplacian of the Gaussian (LOG), where the second derivative of the convolution of the Gaussian kernel and stimulus intensity was derived and is set to zero, reflects the maximum change in intensity at the zero-crossings. This is known as LOG filtering and the result is a Mexican hat function or operator. The image obtained is a binary image where the edges are defined as the zero-crossings between the background and foreground. The Gabor transform is a simulacrum of the cortical visual system. We defined it as the convolution of a Gaussian kernel and a complex planar wave in a spatial domain. Gabor filters can be derived from a mother wavelet by dilation, scaling, or rotation. A large complement of Gabor filters of various scalar values and orientation are convolved with the stimulus function to obtain a preliminary image. To consolidate the picture, we cite an example from nature, a Gabor filter of the planet Saturn (Figure 15) [34-35].

## Conclusions

In conclusion, the phenomenon of visual contrast sensitivity is fundamental for visual edge detection and for providing insight into multi-channel spatial frequency selection of the visual system. Of particular note is that the Fourier transform of a signal provides limited information in the frequency-time domain and is supplanted by wavelet transforms. The uncertainty in signal extraction in the time-frequency domain is minimized by the Fourier transform of the convolution of a Gaussian function and the receptive field function. We have demonstrated how the mathematical convolution of the stimulus function and the filter function accounts for the firing of a neuronal assembly and the formation of a visual percept. On the experimental front, intracellular recordings of visual simple cortical cells concur with a Mexican hat function, an edge detector. Theoretically, the second derivative, the Laplacian of an image, filters out the image and enhances the contour of an image at the zero-crossings. This is also borne out experimentally. The fidelity of an image is governed by the symmetric properties of a Weyl group. The receptive function operator is a unitary operator, preserving the dimensions of an object between the retina and the visual cortex. The mathematical theory is supported by the experimental data, suggesting that simple visual cortical cells are Fourier analyzers and Gabor filters. This review addresses all the fundamental aspects of visual edge detection by the visual cortex. We have attempted to combine theoretical work with experimental data, with an outline of the underlying mathematical foundations.

## Appendices

$\mathrm{I}$ A very basic introduction to Fourier analysis

Any waveform, simple, complex, or discontinuous can be expressed as an infinite sum of sine and cosine waveforms, a series, S(x) [17]. Let us examine an infinite series of sine waves.

$$S(x) = \sum_{n=1}^{\infty } b_{n} sin(nx)$$

The series has three properties:

1. It is periodic 

$$S(x+2\pi ) = S(x)$$

2. It is odd 

$$S(-x) = - S(x)$$

3. It is symmetric 

$$S(0) = S(\pi )$$

Next, we expand our Fourier series into individual terms

$$S(x) = {b_{1}}sin(x) + b_{2}sin(2x) + ... + b{_{k}}sin(kx) + ...$$

The idea now is to extract the $b_{k}$'s. The way to do this is to deploy the orthogonality condition for trigonometric functions. 

$$\int_{0}^{\pi } sin(nx)sin(kx) dx = 0, if n \neq k$$

multiplying both sides by sin(kx) and integrating between 0 and $\pi$

$$\int_{0}^{\pi }sin(kx).S(x) dx = b_{k}\int_{0}^{\pi }sin^{2}(kx)dx$$

The right hand side is an easy integral. Integrating and re-arranging, we arrive at

$$b_{k} = \frac{2}{\pi }\int_{0}^{\pi }S(x).sin(kx) dx$$

. 

The $b_{k}'s$ are the Fourier coefficients.

$\mathrm{I}\mathrm{I}$ Definition of a mathematical group

A group is a set, $G$, with an operation, $\star$, such that when we combine any two elements of a set, we obtain another element within the set [36]. A group has to satisfy four axioms:

1. Closure. For all elements $a,b\in G$, the composition $a\star b\in G$.

2. Associativity. For all elements $a, b, c\in G$, we have $(a\star b)\star c= a\star (b\star c)$.

3. There exists an identity, $e$, such that for all elements $a\in G, a\star e=e\star a$.

4. For each $a \in G$, there exists an inverse $b \in G,$, such that $a \star b = b \star a= e$, the inverse is also written as $a^{-1}$.

We will illustrate the concept of a group with a famous group known as the Klein-4 group, where each element has its own inverse. The Klein-4 group is a symmetry group of a rectangle with the operations of horizontal and vertical reflection and $180^{^{\circ}}$ rotation (Table 5).

**Table 5:** Klein-4 group

*	e	a	b	c
e	e	a	b	c
a	a	e	c	b
b	b	c	e	a
c	c	b	a	e

The group representation is:

$$\left \{ a,b|a^{2}=b^{2}=(ab)^{2}=e \right \}$$

A Lie group is a continuous group that is a differentiable manifold. This means one can perform calculus on the group and Lie groups provide the natural environment for continuous symmetry operations. We can define a Lie group, $G$, on a smooth manifold as a map, $\mathrm{L}$,  such that for all $x,y\in G$:

$$\mathrm{L}:G\times G\rightarrow G;\mathrm{L}(x,y)=xy$$

and

$$x^{-1}y\in G$$

is a smooth map in $G$.

A simple example is the rotation group of matrices, $SO(2)$, where

$$SO(2)=\begin{pmatrix} cos\theta & -sin\theta \\ sin\theta & cos\theta \end{pmatrix}:\theta \in \mathbb{R} \setminus 2\pi \mathbb{Z}$$

$\mathrm{I}\mathrm{I}\mathrm{I}$ ​​​​​​​A simple example of unitarity

Unitary matrices preserve distance. Let us look at the special unitary group, $SU(1)$. Let $r$ be an element of $SU(1)$, then:

$$r^{2} = r^{\dagger }.r\Rightarrow r^{\dagger }=(r^{*})^{T}$$

Then,

$$r^{'} = Rr\Rightarrow(r^{'})^{\dagger }=r^{\dagger }R^{\dagger }$$

Therefore,

$$(r^{'})^{\dagger }r^{'}=r^{\dagger }R^{\dagger }Rr\Rightarrow R^{\dagger }R=I\Rightarrow R^{\dagger }=R^{-1}$$

Therefore, unitary matrices are Hermitian and equal to their own inverses.

Let us return to our receptive field function $\Psi (\omega ,x)=e^{i\omega x}$, which requires two neurons, one for the real component and the other for the complex component, where $\Psi (\omega ,x)=e^{i\omega x}$, where $x$ is the retinal location and $\omega$ is the cortical location.

Let us apply the translation operator:

$$T(f(x))=f(x-t))=e^{-t\frac{\mathrm{d} }{\mathrm{d} x}}f(x)$$

where $f(x)$ is the light intensity at position $x$ in the retina.

A unitary operation means that a translation in the retina corresponds to a similar translation in the visual cortex.

Let $F(\omega )$ be the firing rate of a neuron at a position $\omega$ in the visual cortex, then:

$$F(\omega )=&lt;\Psi (\omega ,x),f(x)>$$

$$F(\omega )=&lt;\Psi (\omega ,x),f(x)>=\int_{-\infty }^{\infty }\Psi^{*} (\omega ,x).f(x)dx$$

We know our receptive field function is:

$$\Psi (\omega ,x)=e^{i\omega x}$$

and we know that:

$$e^{i\omega x}e^{-i\omega x} = I$$

Therefore, our receptive field function is a unitary operator and preserves distances [37].
